# Improving transparency and scientific rigor in academic publishing

**DOI:** 10.1002/cnr2.1150

**Published:** 2018-12-02

**Authors:** Eric M. Prager, Karen E. Chambers, Joshua L. Plotkin, David L. McArthur, Anita E. Bandrowski, Nidhi Bansal, Maryann E. Martone, Hadley C. Bergstrom, Anton Bespalov, Chris Graf

**Affiliations:** ^1^ John Wiley & Sons, Inc. Hoboken New Jersey USA; ^2^ Department of Neurobiology and Behavior Stony Brook University Stony Brook New York USA; ^3^ Department of Neurosurgery David Geffen School of Medicine at UCLA Los Angeles California USA; ^4^ Center for Research in Biological Systems University of California at San Diego San Diego California USA; ^5^ Department of Psychological Science, Program in Neuroscience and Behavior Vassar College Poughkeepsie New York USA; ^6^ Partnership for Assessment and Accreditation of Scientific Practice Heidelberg Germany; ^7^ Valdman Institute of Pharmacology Pavlov First State Medical University St. Petersburg Russia; ^8^ John Wiley & Sons Oxford UK

**Keywords:** Open Science, peer review, policy, publishing, scientific rigor, transparency

## Abstract

Progress in basic and clinical research is slowed when researchers fail to provide a complete and accurate report of how a study was designed, executed, and the results analyzed. Publishing rigorous scientific research involves a full description of the methods, materials, procedures, and outcomes. Investigators may fail to provide a complete description of how their study was designed and executed because they may not know how to accurately report the information or the mechanisms are not in place to facilitate transparent reporting. Here, we provide an overview of how authors can write manuscripts in a transparent and thorough manner. We introduce a set of reporting criteria that can be used for publishing, including recommendations on reporting the experimental design and statistical approaches. We also discuss how to accurately visualize the results and provide recommendations for peer reviewers to enhance rigor and transparency. Incorporating transparency practices into research manuscripts will significantly improve the reproducibility of the results by independent laboratories.

**Significance:**

Failure to replicate research findings often arises from errors in the experimental design and statistical approaches. By providing a full account of the experimental design, procedures, and statistical approaches, researchers can address the reproducibility crisis and improve the sustainability of research outcomes. In this piece, we discuss the key issues leading to irreproducibility and provide general approaches to improving transparency and rigor in reporting, which could assist in making research more reproducible.

## 1 INTRODUCTION

Progress in basic and clinical research is strongly dependent upon asking important research questions, attempting to answer those questions with robust methods, and then communicating the findings. Persuading colleagues that scientific results are objectively obtained and valid involves a willingness to report accurate, robust, and transparent descriptions of the methods, procedures, and outcomes, which will allow for the independent replication, or reproducibility, of those findings (see Box 1 for definitions).

**Box 1. Definitions**

- *Open Science*—the process of making the content and process of producing evidence and claims transparent and accessible to others^41^
- *Methods Reproducibility*—complete and transparent reporting of information required for another researcher to repeat protocols and methods^2^
- *Results reproducibility*—independent attempts to reproduce the same or nearly identical results with the same protocols under slightly different conditions
- *Rigor*—applying the scientific method in the strictest sense to ensure an unbiased experimental design, analysis, interpretation, and reporting of results
- *Transparency*—the process by which the methodology, including the experimental design, data collection, coding, analysis, and tools used in data analysis are clearly visible to all readers
- *Randomization—*the random allocation of participants/subjects to different experimental conditions or the order of sample collection to minimize the possibility of subjective influence in the assignment of subjects or unmeasured variables that might influence the outcome
- *Blinding—*the investigator and study staff are unaware of the group to which the subject was allocated from study onset through data analysis

Publishers have the responsibility of providing a platform for the exchange of scientific information, while at the same time it is the responsibility of the authors, journal editors, and peer reviewers to ensure that the published manuscripts are accurate. While many editors and peer reviewers expect that research published in their journals should be potentially reproducible, there are no set procedures to empirically test whether a finding can be independently reproduced. What's more, other barriers to reproducing results exist, including the laboratory environment, apparatus and test protocols, and animal strain.^1^ A major source of irreproducibility also includes substantial systematic error, which can occur while scientists are conducting the experiments or during statistical analyses.^2^ Systematic error can occur for a variety of reasons, including lack of scientific skill (e.g., two people performing the same experiment may not have the same level of experience) or variability in subject populations or reagents.^3^ In addition, when a researcher has inadequate statistical knowledge or there are honest flaws in the experimental design and statistical output, the errors generated might inappropriately influence the interpretation of the results.^4^, ^5^

Efforts to improve research transparency (and, subsequently, reproducibility) by funders, researchers, and publishers have led to the development of checklists and new author guidelines (see, for example, Cell Press' Structured Transparent Accessible Reporting [STAR] Methods and the *Journal of Neuroscience Research (JNR)* Transparent Science Questionnaire). However, checklists often go unchecked or unenforced by the publishers, editors, and/or peer reviewers^6^ and compliance by the authors is not always wholehearted (M. Macleod personal communication). Publishers cannot always ensure that the results are reproducible, but they can help the authors to present a transparent account of their work, including providing full details of the experimental and statistical procedures and results. Transparent and rigorous accounts of how an experiment was performed, why the authors used specific statistical approaches, and what limitations arise from such work will allow the reviewers, editors, and subsequently readers to better judge the quality of the science.

In this commentary, we offer an update to basic approaches in reporting a thorough account of the experimental design and statistical approaches and provide an overview of data visualization techniques.^7^ It is our hope, as publishers and editors, that these guidelines will help the authors adhere to specific reporting guidelines that promote rigor and transparency in scientific research, which will ensure an accurate and complete account throughout their experiments and discourage publication bias. This, in turn, will promote better, more reproducible science.

## 2 BARRIERS TO REPRODUCIBILITY

Many factors can lead to irreproducibility of scientific results. Oftentimes, these trace back to flaws in the experimental design, statistical analyses (and a lack of understanding of fundamental statistical principles), including low statistical power or inadequate sample sizes, basic reporting of the information essential for labs to independently reproduce results (e.g., biological reagents and reference material), and selective reporting of data/results (e.g., p‐hacking).^4^, ^8^, ^9^ These factors and others might contribute to between 50% and 90% of the published papers being irreproducible.^10^, ^11^, ^12^, ^13^, ^14^, ^15^, ^16^, ^17^ Attempts to reproduce published results costs the United States approximately $28B annually,^9^, ^18^ yet poor descriptions of the published studies lead to a majority of studies becoming non‐replicable.^11^ The next subsections will break down some of the more common barriers to reproducibility.

### 2.1 Neglecting the methods and materials section in manuscripts

The Methods and Materials section of the manuscript is an often neglected area. Journals and authors often limit the methods section to brief descriptions of the procedures or place more complete methods into supplemental materials, or for journals moving away from supplemental material, to online methods that are separate from the article; these are not often critically reviewed by referees and can go unread by the experimenters. Furthermore, reviewers might not be able to adequately review methods and tools and subsequently might fail to notice that key details are missing. This can lead to a lack of complete and transparent reporting of the information required for another researcher to repeat protocols and methods.^2^ Similarly, journals requiring a subsection on statistical analyses rarely ask the authors to provide a full account of the statistical approaches, and the authors may also fail to include a full account of the statistical outputs in the results section. Without a rigorous description of the methods, materials, and statistical approaches, experimenters lack the necessary information to independently replicate or nearly replicate results with the same protocol under similar conditions.^2^, ^13^

### 2.2 Aiming for novelty and impact

Current publication trends place emphasis on the pursuit of novelty and innovation,^19^ which leads to a collection of reporting problems in how data were obtained.^8^ At the most extreme, pressure to publish may lead individuals to rush their experiments, cut corners, make unintentional errors in statistical outputs, or overinterpret the findings,^20^ which can lead to irreproducibility of the scientific findings.

To publish in “high impact” journals, scientists may resort to submitting only their most novel and impactful findings and avoid presenting nonsignificant or incremental findings,^19^ though the latter also have important implications in driving scientific progress. The pressure to publish sensational findings has even led some “high impact” journals to state in their submission forms: “negative results are not accepted”.^21^ This emphasis might encourage scientists to pursue nonlinear lines of investigation in search of statistical significance (e.g., p‐hacking), and may be one driver of scientific misconduct, including falsifying and fabricating data to increase its impact or statistical significance.^5^ At the very least, it leads researchers to omit nonsignificant or incremental findings leading to a bias in the literature, and reinforces the perception that negative findings carry a low priority for publication.^22^, ^23^ This publication bias has led science reporters and the public to declare that it has become more difficult to trust scientific findings.^24^, ^25^

### 2.3 Inadequate training in experimental design, manuscript writing, and reporting tools

Even with the most rigorous reporting guidelines and stringent publication standards, including the precise application of the scientific method to ensure robust and unbiased experimental design, methodology, analysis, interpretation, and reporting of the results,^26^ it is not guaranteed the authors will fully comply. Reporting guidelines cannot overcome poor training in experimental design and statistics, both of which may be responsible for many of the challenges leading to irreproducibility.^27^, ^28^ Indeed, investigators all too often make errors in designing and performing their research, in selecting statistical tests, and in reporting the results.^29^, ^30^ The problem can be exacerbated by errors being passed down by the primary investigator to students, by reviewers not catching these mistakes, and editors not having the expertise to catch specific errors. However, tools to reeducate scientists at all levels in the experimental design and to employ correct data visualization techniques^31^, ^32^ are available (see the National Institutes of Health education modules designed to train students or retrain scientists on the responsible conduct of research, https://www.nih.gov/research‐training/rigor‐reproducibility/training or the National Postdoctoral Association's Responsible Conduct of Research Toolkit). Moreover, many institutions have statistical consultation available to investigators, which should be used; *JNR* and *Brain and Behavior* both hired statistical editors to review the submitted manuscripts for statistical accuracy and *Current Protocols in Neuroscience* recently released a statistical guide that provides general guidelines regarding when, how, and why certain improved statistical techniques might be used in neuroscience research^33^ (see also Motulsky, 2014^34^). These tools helps the authors improve statistical reporting in manuscripts and ensure that the correct approach was used, though statistical reviews may be limited by how much raw data are available.

In addition to the above tools, editorials and commentaries published in various journals attempt to help the authors improve the descriptions of their experimental procedures and results to ensure that the published research is transparently and accurately reported.^35^, ^36^, ^37^, ^38^, ^39^, ^40^ Unfortunately, the authors often fail to incorporate these guidelines into their articles and most journals do not enforce or penalize the authors for not including specific criteria.^6^ Refining the steps necessary to ensure quality control during the peer review and publication processes is essential in order to improve transparency and scientific rigor. Adopting the approaches discussed below will better ensure that the experimental designs are accurate and deviations from that design are explained, with the ultimate goal of increasing the reproducibility of the published data. Journals and publishers should continue to provide detailed guidelines to help the authors during the submission process, but if researchers do not adopt a rigorous and transparent approach to scientific design and reporting from the onset of training, these requirements will continue to fall short.

In the following sections, we outline the key steps to improve transparency and scientific rigor that should be considered during the designing stages of experiments, not just before submission for publication. These requirements can be broadly broken down into (a) reporting criteria to ensure rigor and transparency; (b) transparent account of experimental design; (c) improving statistical rigor and transparency; and (d) peer review to enhance rigor and transparency. Encouraging specific descriptions and a full account of the study will ensure transparency and could improve reproducibility efforts. The next four sections will break down these components to elaborate on how each can improve transparency and rigor in scientific reporting.

## 3 REPORTING CRITERIA TO ENSURE RIGOR AND TRANSPARENCY

The following points describe the key characteristics that must be included in any research design to assess the internal validity, reliability, and potential for reproducibility of scientific findings. Many of these recommendations have been discussed in various venues (e.g., ARRIVE guidelines^7^, ^18^, ^38^, ^41^, ^42^), and some might only be appropriate to specific sciences. However, we feel that inclusion of these criteria, when applicable, into research manuscripts will improve rigor and transparency of the experimental design and statistical approaches.

### 3.1 Appropriately describing the experimental subjects

The methods section of each published study begins with a description of the experimental unit; however, in many cases, the information provided falls short. The experimental units are the entity that is randomly and independently assigned to the treatment conditions (e.g., human subject, animal, littler, cage, fish tank, culture dish, etc.).^43^ The sample size is equal to the number of experimental units. In considering the sample size, one must ensure that the experimental units are independently allocated to the experimental condition, the application of the condition is applied independently to the unit, and the experimental units do not influence one another.^43^ A significant concern in cell biology is determining whether cells or sections, for example, can be considered an experimental unit. In cases where an animal is treated and subsequent testing occurs postmortem (e.g., immunohistochemistry or electrophysiology), then the histological sections, neurons per section, spines per neuron, tumor cells per section etc. are all subsamples of the experimental unit, which is the animal, and should be considered an n of 1.^43^, ^44^ If data are not independent, one strategy is to analyze clustered data (e.g., convert the replicates from a single subject into a single summary statistic.^44^ Alternatively, there are also procedures to accurately model the true variability in data sets using modern statistical techniques (e.g., handling nested data such as cells/animals, littermates).^45^ As Stanley Lazic so eloquently concluded in his recent paper,^46^

...a few simple alterations to a design or analysis can dramatically increase the information obtained without increasing the sample size. In the interest of minimizing animal usage and reducing waste in biomedical research,^15^, ^47^ researchers should aim to maximise power by designing confirmatory experiments around key questions, use focused hypothesis tests, and avoid dichotomising and nesting that ultimately reduce power and provide no other benefits.

An appropriately written section describing the experimental subjects must include a statement of ethical approval (Institutional Review Board approval for human research or Institutional Animal Care and Use Committee approval for animals), followed by the total number of participants involved in each experiment. The authors must also include a clear description of the inclusion and exclusion criteria, which should be prespecified prior to the start of the experiments. Reporting the number of experimental units (i.e., subjects, animals, cells) excluded as well as the reason for exclusion is necessary to prevent the researcher from introducing selection bias that favors positive outcomes and distorts true effects.^48^ Crucially, studies involving human subjects must not reveal individual identifying information but must contain a full description of the participants' demographics as variations in the demographics can lead to confounding variables if not appropriately controlled. When designing an experiment, one must also account for sex as a biological variable (see below). One should carefully review the extant literature to determine whether sex differences might be observed in the study and, if so, design and power the study to test for sex differences. Omitting this step could compromise the rigor of the study.^49^, ^50^

### 3.2 Randomization and blinding procedures

Choices made by investigators during the design and execution of experiments can introduce bias, which may result in the authors reporting false‐positives.^13^, ^39^, ^51^ For example, when investigators are aware of which animals belong to one condition or know that a given treatment should have a specific effect, or human subjects become aware of the conditions they are in, the researchers and participants may inadvertently be biased toward specific findings or alterations in a specific behavior.^52^, ^53^ To reduce bias in subject and outcome selection, the authors should report randomization and blinding procedures.^54^ Implementing and reporting randomization and blinding procedures is simple and can be followed using a basic guide,^52^, ^55^ but to reduce bias, it is essential to report the method of participant randomization to the various experimental groups as well as on random sample processing and collection of data.^38^, ^39^ Moreover, investigators should report whether experimenters are blind to the allocation sequence and also, in animal studies, report whether controls are true littermates of the test group.^44^ Similarly, once the investigator is blind to the conditions, they should remain unaware of the group in which the subject is allocated and the assessment outcome.^39^ Blinding is not always possible. In these cases, procedures to standardize the interventions and outcomes should be implemented and reported so groups are treated as equally as possible. In addition, researchers should consider duplicate assessment outcomes to ensure objectivity.^52^ Attention to reporting these details will reduce bias, avoid mistaking batch effects for treatment effects, and will improve the transparency of how the research was conducted.

### 3.3 Animal housing and husbandry

Many life science disciplines use animal models to test their hypotheses. Few studies provide detailed information regarding housing and husbandry and those reports that contain the information typically do not provide any level of detail that could allow for others to follow similar housing procedures. When using animals, care should be taken to adequately describe the housing and husbandry conditions as these conditions could have profound implications on the experimental results.^56^ At a minimum, the authors should introduce in the abstract the race, sex, species, cell lines, etc. so that the reader will be aware of the population/sample being studied. However, in the methods section, the authors should carefully describe all animal housing and husbandry procedures. For example, it is normally unclear whether animals were single or group housed, and in most journals, the age and/or weight of the animals are commonly omitted.^57^ Other factors that are not commonly reported include information on how the animals were transported from a breeder to the experimenter vivarium (see Good practices in the Transportation of Research Animals, 2006), vivarium temperature, humidity, day/night schedules, how often cages are cleaned, how often animals are handled, whether enrichment is provided in a cage, and cage sizes.^56^ Requiring a full description of housing and husbandry procedures will be essential to the rigor and transparency of the published studies and could help determine why some studies are not reproducible.

### 3.4 Sex as a biological variable

Sex/gender plays an influential role in experimental outcomes. A common practice within research is that findings in one sex (usually males) are generalized to the other sex (usually females). Yet, research consistently demonstrates that sex differences are present across disciplines. For example, as evidence reveals in a recent issue of JNR (see Sex Influences on Nervous System Function), sex not only matters at the macroscopic level, where male and female brains have been found to differ in connectivity,^58^ but at the microscopic level too.^59^ The National Institutes of Health as well as a number of funding agencies mandates the inclusion of sex as a biological variable, yet this mandate is not enforced by most journals. Starting at the study design, the authors must review whether the extant literature suggests that sex differences might be observed in the study, and if so, then design and power the study to test for sex differences. Otherwise, the rigor of the study could be compromised. When publishing the results, the authors must account for sex as a biological variable, whenever possible. At a minimum, the authors should state the sex of the subjects studied in the title and/or abstract of the manuscript. The rationale for choosing only one sex if a single sex study is conducted should also be provided, though discussed as a limitation to the generalizability of the findings. Investigators must also justify excluding either males or females. The assumptions that females are more variable than males or that females must be tested across the estrous cycle are not appropriate as these are not major sources of variability.^60^ This policy is not a mandate to specifically investigate sex differences, but requires investigators to consider sex from the design of the research question through reporting the results.^49^, ^50^ In some instances, sex might not influence the outcomes (e.g.,^61^, ^62^), but balancing sex in animal and cellular models will distinctly inform the various levels of research.^49^ More specific guidelines for applying the policy of considering sex as a biological variable are also available,^50^, ^63^ but shifting the experimental group composition should be done in the context of appropriate *a priori* power analyses. One concern is that sample sizes need to be doubled to identify effects using both female and male subjects, but factorial designs can evaluate the main effects of the treatment and subject sex without increasing the sample size.^64^ While the risk of false‐positive errors associated with testing sex differences in this way is present, reporting that these differences may or may not be present is imperative to understanding how sex influences the function of the nervous system. This practice should be extended to all scientific journals using animal/human subjects.

### 3.5 Transparent account of the experimental design and statistical approaches

A transparent experimental design, meaning how the experiment is planned to meet the specified objectives, describes all the factors that are to be tested in an experiment, including the order of testing and the experimental conditions. As studies become more complex and interconnected, planning the experimental procedures prior to the onset of experiments becomes essential. Yet even when the experiments are planned prior to their initiation, the experimental designs are often poorly described and rarely account for alterations in procedures that were used in the study under consideration. To provide a more transparent and rigorous approach to describing the experimental design, a new section should be placed after the “subjects” paragraph describing, in detail, the experimental design and deviations made from the original design.

The experimental design section should consist of two main components: (a) a list of the experimental procedures that were used to conduct the study, including the sequence and timing of manipulation; and (b) an open discussion of any deviations made from the original design. The description should include an explanation of the question(s) being tested, whether this is a parameter estimation, model comparison, exploratory study, etc., the dependent and independent variables, replicates (how often the experiments were performed and how the data were nested). and the type of design considered (e.g., completely randomized design, randomized complete block design, and factorial design; see^65^, ^66^) for definitions and procedures to implement these designs). Assuming the authors planned the analysis prior to data collection, the authors should describe the specific *a priori* consideration of the statistical methods and planned comparisons^7^ or report that no *a priori* statistical planning was carried out. If the statistical approach deviated from how it was originally designed (see, for example, Registered Reports below), the authors should also report the justification for this change. This open description could help to improve independent research reproducibility efforts and assist reviewers and readers in understanding the rationale for specific approaches.

A precise description of how methodological tools and procedures are prepared and used should also be provided in the experimental design section. Oftentimes, methodological procedures are truncated, forcing the authors to omit critical steps. Alternatively, the authors may report that the methods were previously described but might have modified those procedures without reporting those changes. Due to current publishing constraints, various caveats that go into the methodological descriptions remain unknown. However, this can be remedied easily by journals requiring a full description or step‐by‐step procedure of the experimental protocol used to test the dependent variables. Two options are available for publishing full protocols. First, the protocol could be published in the manuscript, with the reviewers verifying that the procedures are appropriately followed; second, a truncated version of the methods could be published in the manuscript, but the extended methods must be required as supplemental material (the extended methods will be peer reviewed during the submission process). An alternative approach is to deposit step‐by‐step protocols into a database or a data repository such as Dryad, FigShare, or with the Center for Open Science, where they will receive a DOI and can be linked back to the original research article, which will contain the truncated procedures.

#### 3.5.1 Materials

Rigorous descriptions of the experimental protocols not only require a level of detail in the description of the experimental design, but also a full account of the resources and how they were prepared and used. A contributing factor to irreproducibility is the poor or inaccurate description of materials. In order for researchers to replicate and build upon published research findings, they must have confidence in knowing that materials specified in a publication can be correctly identified so that they might obtain the same materials and/or find out more about those materials. Most studies do not include sufficient detail to uniquely identify key research resources, including model organisms, cell lines, and antibodies, to name a few.^67^ While most author guidelines request that the authors provide the company name, city in which the company is located, and the catalog number of the material, (a) many authors do not include this information; (b) the particular product may no longer be available; or (c) the catalog number or lot number is reported incorrectly, thus rendering the materials unattainable.

A new system is laying the foundation to report research resources with a unique identification number that can be deposited in a database for quick access. The Resource Identification Initiative standardizes the materials necessary to conduct research by assigning research resource identifiers (RRIDs).^68^ To make it as simple as possible to obtain RRIDs, a platform was developed (www.scicrunch.org/resources) to aggregate data about antibodies, cell lines, model organisms, and software into a community database that is automatically updated on a weekly basis and provides the most recent articles that contain RRIDs. While SciCrunch is among the founding platforms, these identifiers can also be found on other sites, including antibodyregistry.org, benchsci.com, and others. Similarly, though more involved, PubChem offers identification for various compounds such as agonists and antagonists. Simply find the chemical abstract service (CAS) number from the chemical safety data sheet (SDS), input that number into PubChem, and receive the PubChem Chemical Identifier (CID). RRIDs have been successfully implemented in many titles throughout Wiley and are also in use by Cell Press and a number of other publishers. The authors should provide RRIDs and CIDs when describing resources such as antibodies, software (including statistical software used, as this is rarely reported), and model organisms, or compounds used, allowing for easy verification by peer reviewers and experimenters.

#### 3.5.2 Statistical rigor and transparency

With most statistical software having a user‐friendly interface, students quickly learn how to perform basic statistical tests. However, users all too often choose inadequate and incorrect statistical methods or approaches or cannot reproduce their analyses since they have only a rudimentary understanding to each test and when to use them.^6^, ^28^, ^69^, ^70^ What's more, the authors do not appropriately describe their statistical approaches in text, partially because tests are performed only after the study is executed. In designing and reporting the experiments, the authors should report normalization procedures, tests for assumptions, exclusion criteria, and why statistical approaches might differ from what the authors originally proposed, if they developed these approaches prior to the onset of data collection. In addition, the authors must also include the statistical software and specific version thereof, descriptive statistics, and a full account of the statistical outputs in the results section.

Errors in statistical outputs often arise when the authors (a) do not conduct and report a power calculation^70^ or do not distinguish between exploratory and confirmatory analyses;^71^ (b) fail to state which statistical tests are used or provide adequate detail about the tests, including the descriptive statistics and a full account of the statistical output; (c) fail to state whether assumptions were examined^42^; or (d) fail to describe how replicates were analyzed.^69^ Moreover, it might be difficult to reproduce statistical output when the authors do not report the statistical software and specific version thereof, fail to include in the manuscript the exclusion criteria or code used to generate analyses, or explain how modifications to the experimental design might lead to changes in how statistical analyses are approached (e.g., independent versus non‐independent groups) (additional details about these common mistakes can be found in,^7^, ^28^, ^32^ but it is important to emphasize that failure to report these variables can lead to errors in data interpretation.

Choosing the correct statistical analyses first depends on an appropriate experimental design and mode of investigation (exploratory versus confirmatory^71^). One must decide whether experimental conditions are independent, meaning that no subjects or specimens are related to each other,^7^, ^32^ whether the conditions are non‐independent or paired, and whether there are any associations between variables.^72^ The second step is that statistical analyses must include specific details about the test statistics, rationale for choosing each test, a description of whether normal distribution parameters are obtained, and a statement about which p‐value level is deemed statistically significant. In addition, a transparent and rigorous statistical analysis section must include the following:

- Power analysis calculations or sample size justification for exploratory research, including accuracy in parameter estimation^73^
- Statement of the factors tested, types of analyses, and what post hoc comparisons were made
- Statement of the statistical tests used and details as to why those tests were chosen, including how the authors choose between parametric or nonparametric tests (assumptions aside)*
- Statement of an assessment of assumptions
- Statement of how replicates were analyzed (e.g., are western blots performed in duplicate and band pixels averaged?)
- Data point exclusion criteria
- Statement of how outliers were determined and how they were handled
- Descriptions of raw data, including transformation procedures
- Within the results, a full account of the test statistic, and where applicable the degrees of freedom, p‐values reported to a consistent number of decimal places (usually three), and statement of whether the test was one‐ or two‐sided

#### 3.5.3 Power analysis

Many studies are rejected for publication because of criticism that a study is underpowered, though many more studies are published despite this.^74^ Reporting how a sample size was predetermined based on power analyses conducted during the experimental design stage is a good way to avoid this criticism. Researchers are taught to perform these analyses prior to the start of their experiments, but evidence suggests that researchers and peer reviewers do not fully understand the concept of statistical power, have not been given adequate education about the concept, or do not consider the measurement important in designing the experiments.^75^

Reviewers and journal editors are beginning to ask authors to address the question of what the power of the study was to detect the observed effect.^76^, ^77^ Determining whether a study is appropriately powered *a priori* or *post hoc* is a matter of debate.^77^ Many argue that *post hoc* power analyses are inappropriate, especially for nonsignificant findings, while others argue that *post hoc* power analyses are appropriate since *a priori* power analyses do not represent the power of the ensuring effect, but rather the hypothesized effect.^75^

The *a priori* power analysis is the most common way of determining the sample size for simple experiments and can be easily computed using freely available software such as G*Power. The sample size depends on a mathematical relationship among the (a) effect size of interest; (b) standard deviation (*SD*); (c) chosen significance level; (d) chosen power; and (e) alternative hypothesis.^54^ Yet, as more parameters come into play (for example, within mixed effects modeling), power analysis software becomes more complex (see Power Analysis for Mixed Effect Models in R). Conducting these analyses allows researchers to confidently select a sample size large enough to lead to a rejection of the null hypothesis for a given effect size.^75^ However, one limitation to *a priori* power analyses is that effect sizes and *SD*s may not be known prior to the research being conducted and may lead to observed effects that are smaller or larger than the hypothesized effects,^78^, ^79^). Alternatively, if it is conventional to use a specific number of subjects for a particular test, then one can report the calculated effect size for that particular sample size and decide whether more samples would be warranted. Either way, power and sample size calculations provide a single estimate, ignoring variability and uncertainty as such simulations are highly encouraged (see^80^).

An alternative to the *a priori* power analysis is a *post hoc* power analysis (SPSS calls this “observed power”) or confidence intervals. The *post hoc* power analysis takes the observed effect size as the assumed population effect, though this computation might be different from a true population effect size, which might culminate in a misleading evaluation of power.^75^ *Post hoc* power analyses always show there is low power with respect to nonsignificant findings.^77^ Thus, utilizing the *post hoc* power analysis must be done with extreme care and should never be a substitute for the *a priori* power analysis. In fact, many in the statistical community see *post hoc* analyses as a waste of effort and recommend abandoning this approach^81^; see also https://dirnagl.com/2014/07/14/why‐post‐hoc‐power‐calculation‐does‐not‐help/ and http://daniellakens.blogspot.com/2014/12/observed‐power‐and‐what‐to‐do‐if‐your.html). If a reviewer or journal requests a power analysis, we recommend that rather than using *post hoc* power analyses, report confidence intervals to estimate the magnitude of effects that are consistent with the statistical data reported.^76^, ^77^, ^82^ Alternatively, if increasing power is a necessity and/or sample sizes are already at their limits for financial or logistic reasons, one should consider alternative approaches, which are well described by Lazic; these include: (a) using fewer factor values for continuous predictors; (b) having a more focused and specific hypothesis test; (c) not dichotomizing or binning continuous variables; (d) using a crossed or factorial design rather than a nested arrangement.^46^

We also advise authors to determine whether a parametric or nonparametric test is the most appropriate for the obtained data. Analogues to ordinary parametric tests (e.g., *t*‐test or ANOVA, etc.) can be performed even if data are skewed or have nonnormal distributions; multiple robust analytics are available for these circumstances (see^83^) as long as the sample size is sufficient. Importantly, parametric tests also generally have somewhat more statistical power than nonparametric tests and are more likely to detect a significant effect if one exists. Alternatively, when one's data are better represented by the median, nonparametric tests may be more appropriate, especially when data are skewed enough that a mean might be strongly affected by the distribution tail, whereas the median estimates the center of the distribution. Nonparametric tests may also be more appropriate when the obtained sample size is small, as occurs in many fields where sample sizes average less than eight per group^48^ or when the data obtained are ordinal, ranked, or there are outliers that cannot be removed.^84^ Beware, however, that meaningful nonparametric testing with sample sizes too low (e.g., *n* < 5) contains very little appreciable power to reveal an effect, if indeed one is present; difficulties due to violations of the underlying statistical assumptions of the particular test being used might be present. Bayesian analyses with small sample sizes are also possible, though estimates are highly sensitive to the specification of the prior distribution.

#### 3.5.4 Graphical representation of data

Figures illustrate the most important findings from a study by conveying information about the study design in addition to showing the data and statistical outputs.^7^, ^32^ Simplistic representations to visualize the data are commonly used and are often inappropriate. For example, bar graphs are designed for categorical data; when used to display continuous data, bar graphs with error bars omit key information about the data distribution (see also^85^). To change standard practices for presenting data, continuous data should be visualized by emphasizing the individual points; dot plots (e.g., univariate scatterplots) are strongly recommended for small samples, along with plots such as violin plots (or overlaid points on the plots) to provide far more informative views of the data distributions when samples are sufficiently large. Bar graphs should be reserved for categorical data only. Moreover, graphic data plots involving multiple groups are often shown as overlaid, but should be “jittered” across the *X*‐axis so that each discrete data point can be visualized. The use of jittering means that when there are fewer unique combinations of data points than total observations, the totality of the data distribution is not obscured. By adopting these practices, readers will be better able to detect gross violations of the statistical assumptions and determine whether results would be different using alternate strategies.^42^

When plotting data, it is important to also report the variability of the data. Typically, this is expressed as the *SD* or standard error of the mean (*SEM*), but it is important to note that *SEM* does indicate variability.^34^ The *SD* is calculated as part of an estimate of the variability of the population from which the sample was drawn.^86^, ^87^ The *SEM*, on the other hand, describes the *SD* of the sample mean as an estimate of the accuracy of the population mean. In other words, the *SD* shows how many points within the sample differ from the sample mean, whereas the *SEM* shows how close the sample mean is to the population mean.^87^ The main function of *SEM* is to help construct confidence intervals, which are a range of values that take into account the true population value (usually an unknown), so that one can quantify the proximity of the experimental mean to the population mean.^88^ Yet deriving confidence intervals around one's data (using *SD*) or the mean (using *SEM*) is premised on those data being normally distributed. Robust estimators are increasingly important as heteroscedasticity (having subpopulations with differing variabilities) is a frequent consequence of real‐world measurement. Traditional data transformations are an attempt to cope with this phenomenon but for many, such transformations may not actually serve to resolve anything and may add a layer of unnecessary complexity.

In determining which estimate of variability to depict graphically, it is important to remember that the *SD* is used when one wants to know how widely scattered measurements are or the variability within the sample, but if one is interested in the uncertainty around the estimate of the mean measurement or the proximity of the mean to the population mean, *SEM* is more appropriate.^87^ When plotting data variability, it is important to consider that when *SEM* bars do not overlap, the viewer cannot be sure that the difference between the two means is statistically significant (see^34^). We also note that it is misleading to report *SD*'s in the narrative and tables but plot *SEM*s. Furthermore, unless an author specifically wants to inform the reader about the precision of the study, *SD* should be reported as it quantifies variability within the sample.^86^, ^87^, ^88^ Therefore, the optimal method to visualize data variability is to display the raw data, but if that makes the graph too difficult to read, instead show a box‐whisker plot, frequency distribution, or the mean ± *SD*.^34^

#### 3.5.5 Inclusion of statistically significant and nonsignificant data

The probability that a scientific research article is published traditionally depends on the novelty or inferred impact of the conclusion, the size of the effect measured, and the statistical confidence in that result.^21^, ^89^ The consequence of obtaining negative results can lead to a file‐drawer effect; scientists ignore negative evidence that does not reach significance and intentionally or unintentionally select the subsets of data that show statistical significance as the outcomes of interest.^41^ This publication bias skews scientific knowledge toward statistically significant or “positive” results, meaning that the results of thousands of experiments that fail to confirm a result are filed away.^89^ These data‐contingent analysis decisions, also known as p‐hacking,^90^ can inflate spurious findings and lead to misestimates that might have consequences for public health. To combat the stigma of reporting negative results, we encourage authors to provide a full account of the experiment, to explicitly state both statistically significant and nonsignificant results, and to publish papers that have been rigorously designed and conducted, irrespective of their statistical outcomes. In addition, some organizations such as the European College of Neuropsychopharmacology are offering prizes in neuroscience research to encourage publication of data where the results do not confirm the expected outcome or original hypothesis (see ECNP Preclinical Network Data Prize). Published reports of both significant and nonsignificant findings will result in better scientific communication among and between colleagues.

#### 3.5.6 Real and perceived conflicts of interest

Though objectivity of a researcher or group is assumed, conflicts of interest may exist and could be a potential source of bias. Conflicts of interest largely focus on financial conflicts,^91^, ^92^ but they can also occur when an individual's personal interests are in conflict with professional obligations, including industrial relationships.^93^ Conflicts, whether real or perceived, arise when one recognizes an interest as influencing an author's objectivity. This can occur when an author owns a patent, or has stock ownership, or is a member of a company, for example. All participants in a paper must disclose all relationships that could be viewed as presenting a real or perceived conflict of interest. When considering whether a conflict is present, one should ask whether a reasonable reader could feel misled or deceived. While beyond the scope of this article, the Committee on Publication Ethics offers a number of resources on conflicts of interest.

#### 3.5.7 Registered reports and open practices badges

One possible way to incorporate all the information listed above and to combat the stigma against papers that report nonsignificant findings is through the implementation of Registered Reports or rewarding transparent research practices. Registered Reports are empirical articles designed to eliminate publication bias and incentivize best scientific practice. Registered Reports are a form of empirical article in which the methods and the proposed analyses are preregistered and reviewed prior to research being conducted. This format is designed to minimize bias, while also allowing complete flexibility to conduct exploratory (unregistered) analyses and report serendipitous findings. The cornerstone of the Registered Reports format is that the authors submit as a Stage 1 manuscript an introduction, complete and transparent methods, and the results of any pilot experiments (where applicable) that motivate the research proposal, written in the future tense. These proposals will include a description of the key research question and background literature, hypotheses, experimental design and procedures, analysis pipeline, a statistical power analysis, and full description of the planned comparisons. Submissions, which are reviewed by editors, peer reviewers and in some journals, statistical editors, meeting the rigorous and transparent requirements for conducting the research proposed are offered an in‐principle acceptance, meaning that the journal guarantees publication if the authors conduct the experiment in accordance with their approved protocol. Many journals publish the Stage 1 report, which could be beneficial not only for citations, but for the authors' progress reports and tenure packages. Following data collection, the authors prepare and resubmit a Stage 2 manuscript that includes the introduction and methods from the original submission plus their obtained results and discussion. The manuscript will undergo full review; referees will consider whether the data test the authors' proposed hypotheses by satisfying the approved outcome‐neutral conditions, will ensure the authors adhered precisely to the registered experimental procedures, and will review any unregistered *post hoc* analyses added by the authors to confirm they are justified, methodologically sound, and informative. At this stage, the authors must also share their data (see also Wiley's Data Sharing and Citation Policy) and analysis scripts on a public and freely accessible archive such as Figshare and Dryad or at the Open Science Framework. Additional details, including template reviewer and author guidelines, can be found by clicking the link to the Open Science Framework from the Center for Open Science (see also^94^).

The authors who practice transparent and rigorous science should be recognized for this work. Funders can encourage and reward open practice in significant ways (see https://wellcome.ac.uk/what‐we‐do/our‐work/open‐research). One way journals can support this is to award badges to the authors in recognition of these open scientific practices. Badges certify that a particular practice was followed, but do not define good practice. As defined by the Open Science Framework, three badges can be earned. The Open Data badge is earned for making publicly available the digitally shareable data necessary to reproduce the reported results. These data must be accessible via an open‐access repository, and must be permanent (e.g., a registration on the Open Science Framework, or an independent repository at www.re3data.org). The Open Materials badge is earned when the components of the research methodology needed to reproduce the reported procedure and analysis are made publicly available. The Preregistered badge is earned for having a preregistered design, whereas the Preregistered+Analysis Plan badge is earned for having both a preregistered research design and an analysis plan for the research; the authors must report results according to that plan. Additional information about the badges, including the necessary information to be awarded a badge, can be found by clicking this link to the Open Science Framework from the Center for Open Science.

## 4 PEER REVIEW TO ENHANCE RIGOR AND TRANSPARENCY

The process of peer review is designed to evaluate the validity, quality, and originality of the articles for publication. Yet peer reviewers are not immune to making mistakes. For example, several studies were conducted where major errors were inserted into papers. In these studies, no reviewer ever found all the errors and some reviewers did not spot any errors.^95^, ^96^ While it is beyond the scope of this article to discuss many of the defects of peer review (see^97^), it is important to note that the changes to the peer review process are ongoing^98^ and publishers are working to develop more formal training processes. However, to quickly improve rigor and transparency in scientific research, peer review should emphasize the design and execution of the experiment. We are not saying that reviewers should focus solely on the experimental design; it is important for reviewers to weigh in on the novel insights of a study and how study results may or may not contribute to the field. However, to help ensure the accuracy and the validity of a study, emphasis should first be on the experimental design. To assist the reviewers, the authors should submit as part of their manuscript a Transparent Science Questionnaire (TSQ), or something equivalent, which identifies where in the manuscript specific elements that could aid in reproducibility efforts are found. The reviewers use this form to verify that the authors have included the relevant information and ensure that the study was designed and executed objectively, ensuring the study's validity and reliability. Using this or similar forms will also help reviewers to find the relevant information necessary to ensure the appropriateness of the design, which can then allow them to focus on the experimental outcomes. Adopting forms such as the TSQ or using services such as those offered by Research Square could also speed up the peer review process and reduce the cost in time committed by unpaid reviewers (which, in 2008, was estimated to cost $2.3 billion) (https://scholarlykitchen.sspnet.org/2010/08/31/the‐burden‐of‐peer‐review/).

A multistage review where different parties are concerned with different aspects of the review may be optimal. Because many errors in manuscripts are found in the statistical output, one stage of review should be a statistical review, whereby a statistical editor reviews the statistical analyses of the manuscript to ensure accuracy, but also verifies that the most appropriate statistical tests for that design were used. Upon completion, the editor will then make a decision as to whether the approach and execution is sufficient and is in line with the reported statistical output. By having experts focus on specific aspects of a research report, journal editors will become more confident that the research published is valid and of high quality and integrity.

## 5 CONCLUSIONS

A challenge in science is for scientists to be open and transparent about the procedures used to obtain results. A major source of irreproducibility is substantial human error, which can occur while scientists are conducting the experiments or during data/statistical analysis. Groups are continuing to develop systems that help researchers cover every aspect of the experimental design (e.g., EQIPD or XDA), but education and awareness of the key elements in research design and analysis is essential to transparent and reproducible research. By incorporating the specific elements discussed in this document into research manuscripts, researchers can reduce subjective bias, while actively improving methods' reproducibility, which will increase the likelihood of research reproducibility as the two are closely linked.^2^ While variability in results is inevitable, ensuring that every salient aspect of a study is reported will help others understand the procedures involved and potential sources of errors during the experimentation process, which will ultimately lead to greater transparency in science.

## CONFLICT OF INTEREST

Dr. David McArthur serves as JNR's paid statistical reviewer and has reviewed in that capacity for other journals, both Wiley and other publishers. Dr. Anita Bandrowski runs SciCrunch, a company devoted to ensuring RRIDs persist in the literature. Dr. Maryann Martone is a founder and the CSO of SciCrunch, which provides services supporting RRIDs and is the Editor‐in‐Chief of *Brain and Behavior*. Dr. Eric Prager is the Editor‐in‐Chief of *Journal of Neuroscience Research*. Dr. Nidhi Bansal is the Editor‐in‐Chief of *Cancer Reports*. Chris Graf works for Wiley, and volunteers for COPE, Committee on Publication Ethics.

## AUTHORS' CONTRIBUTION

All authors take responsibility for the integrity and the accuracy of this manuscript. *Conceptualization*, EMP and CG. *Writing—Original Draft*, EMP, KC; *Writing—Review and Editing*, EMP, KC, JKP, DLM, AB, NB, MM, HCB, AB, CG; *Supervision*, CG.

## Supporting information

A preprint of this paper, which includes a roadmap to follow when preparing original research manuscripts and comments made during the review of the paper can be found at https://osf.io/5cvqh/.

## ENDNOTE

*
When describing the data, it is important to differentiate between an exploratory and confirmatory study, as this could have profound implications as to how data are presented. Exploratory analyses are meant to identify patterns in the data without much emphasis on hypothesis testing, but most studies publish confirmatory experiments to test one or a few stated hypotheses.

## References

[1] Crabbe JC , Wahlsten D , Dudek BC . Genetics of mouse behavior: interactions with laboratory environment. Science. 1999;284(5420):1670‐1672. 10356397 10.1126/science.284.5420.1670

[2] Goodman SN , Fanelli D , Ioannidis JP . What does research reproducibility mean? Sci Transl Med. 2016;8(341):341ps312. 10.1126/scitranslmed.aaf5027 27252173

[3] Capes‐Davis A , Neve RM . Authentication: a standard problem or a problem of standards? PLoS Biol. 2016;14(6):e1002477. 10.1371/journal.pbio.1002477 27300550 PMC4907433

[4] Baker M . Is there a reproducibility crisis? Nature. 2016;533(7604):452‐454. 27225100 10.1038/533452a

[5] Steen RG , Casadevall A , Fang FC . Why has the number of scientific retractions increased? PLoS One. 2013;8(7):e68397. 10.1371/journal.pone.0068397 23861902 PMC3704583

[6] Baker D , Lidster K , Sottomayor A , Amor S . Two years later: journals are not yet enforcing the ARRIVE guidelines on reporting standards for pre‐clinical animal studies. PLoS Biol. 2014;12(1):e1001756. 10.1371/journal.pbio.1001756 24409096 PMC3883646

[7] Weissgerber TL , Garovic VD , Winham SJ , Milic NM , Prager EM . Transparent reporting for reproducible science. J Neurosci Res. 2016;94(10):859‐864. 10.1002/jnr.23785 27377972 PMC5330667

[8] Forstmeier W , Wagenmakers EJ , Parker TH . Detecting and avoiding likely false‐positive findings—a practical guide. Biol Rev Cambridge Philo Soc. 2017;92(4):1941‐1968. 10.1111/brv.12315 27879038

[9] Freedman LP , Cockburn IM , Simcoe TS . The economics of reproducibility in preclinical research. PLoS Biol. 2015;13(6):e1002165. 10.1371/journal.pbio.1002165 26057340 PMC4461318

[10] Begley CG , Ellis LM . Drug development: Raise standards for preclinical cancer research. Nature. 2012;483(7391):531‐533. 10.1038/483531a 22460880

[11] Glasziou P , Altman DG , Bossuyt P , et al. Reducing waste from incomplete or unusable reports of biomedical research. The Lancet. 2014;383(9913):267‐276. 10.1016/S0140-6736(13)62228-X 24411647

[12] Hartshorne JK , Schachner A . Tracking replicability as a method of post‐publication open evaluation. Front Comp Neurosci. 2012;6:8. 10.3389/fncom.2012.00008 PMC3293145 22403538

[13] Kilkenny C , Parsons N , Kadyszewski E , et al. Survey of the quality of experimental design, statistical analysis and reporting of research using animals. PLoS One. 2009;4(11):e7824. 10.1371/journal.pone.0007824 19956596 PMC2779358

[14] Macleod MR , Lawson McLean A , Kyriakopoulou A , et al. Risk of bias in reports of in vivo research: a focus for improvement. PLoS Biol. 2015;13(10):e1002273. 10.1371/journal.pbio.1002273 26460723 PMC4603955

[15] Macleod MR , Michie S , Roberts I , et al. Biomedical research: increasing value, reducing waste. Lancet. 2014;383(9912):101‐104. 10.1016/S0140-6736(13)62329-6 24411643

[16] Moher D , Altman DG . Four proposals to help improve the medical research literature. PLoS Med. 2015;12(9):e1001864. 10.1371/journal.pmed.1001864 26393914 PMC4579130

[17] van der Worp HB , Macleod MR . Preclinical studies of human disease: time to take methodological quality seriously. J Mol Cell Cardiol. 2011;51(4):449‐450. 10.1016/j.yjmcc.2011.04.008 21549125

[18] Freedman LP , Venugopalan G , Wisman R . Reproducibility2020: Progress and priorities. F1000Research. 2017;6:604. 10.12688/f1000research.11334.1 28620458 PMC5461896

[19] Cohen BA . How should novelty be valued in science? Elife. 2017;6:e28699. 10.7554/eLife.28699 28742499 PMC5526661

[20] Alberts B , Kirschner MW , Tilghman S , Varmus H . Rescuing US biomedical research from its systemic flaws. Proc Natl Acad Sci. 2014;111(16):5773‐5777. 10.1073/pnas.1404402111 24733905 PMC4000813

[21] Matosin N , Frank E , Engel M , Lum JS , Newell KA . Negativity towards negative results: Adiscussion of the disconnect between scientific worth and scientific culture. Dis Model Mech. 2014;7(2):171‐173. 10.1242/dmm.015123 24713271 PMC3917235

[22] Capuano A , Coats AJ , Scavone C , Rossi F , Rosano GM . Disclosure of negative trial results. A call for action. Int J Cardiol. 2015;198:47‐48. 10.1016/j.ijcard.2015.06.157 26151710

[23] Dickersin K , Min YI , Meinert CL . Factors influencing publication of research results. Follow‐up of applications submitted to two institutional review boards. JAMA. 1992;267(3):374‐378. 1727960

[24] Bosman J . Reporters find science journals harder to trust, but not easy to verify. New York Times; 2006. 16514742

[25] Laine C , Goodman SN , Griswold ME , Sox HC . Reproducible research: moving toward research the public can really trust. Ann Intern Med. 2007;146(6):450‐453. 17339612 10.7326/0003-4819-146-6-200703200-00154

[26] Rigor and Reproducibility . 2017. Retrieved from https://grants.nih.gov/reproducibility/index.htm

[27] Collins FS , Tabak LA . Policy: NIH plans to enhance reproducibility. Nature. 2014;505(7485):612‐613. 24482835 10.1038/505612a PMC4058759

[28] Weissgerber TL , Garovic VD , Milin‐Lazovic JS , et al. Reinventing biostatistics education for basic scientists. PLoS Biol. 2016;14(4):e1002430. 10.1371/journal.pbio.1002430 27058055 PMC4825954

[29] Steward, O. A rhumba of "R's": replication, reproducibility, rigor, robustness: what does a failure to replicate mean? eNeuro, 2016;3(4): 1–4. 10.1523/ENEURO.0072-16.2016 PMC4937130 27419220

[30] Yamada KM , Hall A . Reproducibility and cell biology. J Cell Biol. 2015;209(2):191‐193. 10.1083/jcb.201503036 25918221 PMC4411270

[31] Weissgerber TL , Garovic VD , Savic M , Winham SJ , Milic NM . From static to interactive: transforming data visualization to improve transparency. PLoS Biol. 2016;14(6):e1002484. 10.1371/journal.pbio.1002484 27332507 PMC4917243

[32] Weissgerber TL , Savic M , Winham SJ , Stanisavljevic D , Garovic VD , Milic NM . Data visualization, bar naked: a free tool for creating interactive graphics. J Biol Chem. 2017;292(50):20592‐20598. https://doi.org/10.1074/jbc. RA117.000147 28974579 10.1074/jbc.RA117.000147 PMC5733595

[33] Wilcox RR , Rousselet GA . A guide to robust statistical methods in neuroscience. Current Protocols in Neuroscience *,* 2018;82:8. 10.1002/cpns.41 29357109

[34] Motulsky HJ . Common misconceptions about data analysis and statistics. J Pharmacolo Experiment Therap. 2014;351(1):200‐205. 10.1124/jpet.114.219170 25204545

[35] Bravo E , Calzolari A , De Castro P , et al. Developing a guideline to standardize the citation of bioresources in journal articles (CoBRA). BMC Med. 2015;13(1):33. 10.1186/s12916-015-0266-y 25855867 PMC4331335

[36] Collins GS , Reitsma JB , Altman DG , Moons KG . Transparent reporting of a multivariable prediction model for individual prognosis or diagnosis (TRIPOD). Ann Intern Med. 2015;162(10):735‐736. 10.7326/L15-5093-2 25984857

[37] Hooijmans CR , Leenaars M , Ritskes‐Hoitinga M . A gold standard publication checklist to improve the quality of animal studies, to fully integrate the three Rs, and to make systematic reviews more feasible. Altern Lab Anim. 2010;38(2):167‐182. 20507187 10.1177/026119291003800208

[38] Kilkenny C , Browne WJ , Cuthill IC , Emerson M , Altman DG . Improving bioscience research reporting: the ARRIVE guidelines for reporting animal research. PLoS Biol. 2010;8(6):e1000412. 10.1371/journal.pbio.1000412 20613859 PMC2893951

[39] Landis SC , Amara SG , Asadullah K , et al. A call for transparent reporting to optimize the predictive value of preclinical research. Nature. 2012;490(7419):187‐191. 10.1038/nature11556 23060188 PMC3511845

[40] Shamseer L , Moher D , Clarke M , et al. Preferred reporting items for systematic review and meta‐analysis protocols (PRISMA‐P) 2015: elaboration and explanation. BMJ. 2015;349(jan02 1):g7647. 10.1136/bmj.g7647 25555855

[41] Munafo MR , Nosek BA , Bishop DVM , et al. A manifesto for reproducible science. Nat Hum Behav. 2017;1(0021):1‐9. 10.1038/s41562-016-0021 PMC7610724 33954258

[42] Weissgerber TL , Milic NM , Winham SJ , Garovic VD . Beyond bar and line graphs: time for a new data presentation paradigm. PLoS Biol. 2015;13(4):e1002128. 10.1371/journal.pbio.1002128 25901488 PMC4406565

[43] Lazic SE , Clarke‐Williams CJ , Munafo MR . What exactly is 'N' in cell culture and animal experiments? PLoS Biol. 2018;16(4):e2005282. 10.1371/journal.pbio.2005282 29617358 PMC5902037

[44] Galbraith S , Daniel JA , Vissel B . A study of clustered data and approaches to its analysis. J Neurosci. 2010;30(32):10601‐10608. 10.1523/JNEUROSCI.0362-10.2010 20702692 PMC6634702

[45] Wilson MD , Sethi S , Lein PJ , Keil KP . Valid statistical approaches for analyzing sholl data: mixed effects versus simple linear models. J Neurosci Methods. 2017;279:33‐43. 10.1016/j.jneumeth.2017.01.003 28104486 PMC5346342

[46] Lazic SE . Four simple ways to increase power without increasing the sample size. Lab Anim. 2018;23677218767478:002367721876747. 10.1177/0023677218767478 29629616

[47] Ioannidis JP , Greenland S , Hlatky MA , et al. (2014). Increasing value and reducing waste in research design, conduct, and analysis. Lancet *,* 383(9912):11–17, 166–175. 10.1016/S0140-6736(13)62227-8 PMC4697939 24411645

[48] Holman C , Piper SK , Grittner U , et al. Where have all the rodents gone? The effects of attrition in experimental research on cancer and stroke. PLoS Biol. 2016;14(1):e1002331. 10.1371/journal.pbio.1002331 26726833 PMC4699644

[49] Clayton JA . Studying both sexes: Aguiding principle for biomedicine. FASEB J. 2016;30(2):519‐524. 10.1096/fj.15-279554 26514164 PMC4714546

[50] Clayton JA . Applying the new SABV (sex as a biological variable) policy to research and clinical care. Physiol Behav. 2018;187:2‐5. 10.1016/j.physbeh.2017.08.012 28823546

[51] Kilkenny C , Browne W , Cuthill IC , Emerson M , Altman DG , Group, N. C. R. R. G. W . Animal research: reporting in vivo experiments: the ARRIVE guidelines. Br J Pharmacol. 2010;160(7):1577‐1579. 10.1111/j.1476-5381.2010.00872.x 20649561 PMC2936830

[52] Karanicolas PJ , Farrokhyar F , Bhandari M . Practical tips for surgical research: blinding: who, what, when, why, how? Can J Surg. 2010;53(5):345‐348. 20858381 PMC2947122

[53] Schulz KF , Grimes DA . Blinding in randomised trials: hiding who got what. Lancet. 2002;359(9307):696‐700. 10.1016/S0140-6736(02)07816-9 11879884

[54] Festing MF , Altman DG . Guidelines for the design and statistical analysis of experiments using laboratory animals. ILAR J. 2002;43(4):244‐258. 12391400 10.1093/ilar.43.4.244

[55] Smith P , Morrow R , Ross D . Randomization, blinding and coding. In: Smith P , Morrow R , Ross D , eds. Field trials of health interventions: A toolbox. 3rd ed. London, UK: Oxford University Press; 2015:1‐23. 26225404

[56] Prager EM , Bergstrom HC , Grunberg NE , Johnson LR . The importance of reporting housing and husbandry in rat research. Front Behav Neurosci. 2011;5:38. 10.3389/fnbeh.2011.00038 21847375 PMC3147140

[57] Florez‐Vargas O , Brass A , Karystianis G , et al. Bias in the reporting of sex and age in biomedical research on mouse models. Elife. 2016;5:e13615. 10.7554/eLife.13615 26939790 PMC4821800

[58] Ingalhalikar M , Smith A , Parker D , et al. Sex differences in the structural connectome of the human brain. Proc Natl Acad Sci USA. 2014;111(2):823‐828. 10.1073/pnas.1316909110 24297904 PMC3896179

[59] Jazin E , Cahill L . Sex differences in molecular neuroscience: From fruit flies to humans. Nat Rev Neurosci. 2010;11(1):9‐17. 10.1038/nrn2754 20019686

[60] Beery AK . Inclusion of females does not increase variability in rodent research studies. Curr Opin Behav Sci. 2018;23:143‐149. 30560152 10.1016/j.cobeha.2018.06.016 PMC6294461

[61] Fritz AK , Amrein I , Wolfer DP . Similar reliability and equivalent performance of female and male mice in the open field and water‐maze place navigation task. Am J Med Genet C Semin Med Genet. 2017;175(3):380‐391. 10.1002/ajmg.c.31565 28654717 PMC5638061

[62] Segarra I , Modamio P , Fernandez C , Marino EL . Sex‐divergent clinical outcomes and precision medicine: an important new role for institutional review boards and research ethics committees. Front Pharmacol. 2017;8:488. 10.3389/fphar.2017.00488 28785221 PMC5519571

[63] McCarthy MM , Woolley CS , Arnold AP . Incorporating sex as a biological variable in neuroscience: what do we gain? Nat Rev Neurosci. 2017;18(12):707‐708. 10.1038/nrn.2017.137 29097784

[64] Collins LM , Dziek JJ , Kugler KC , Trall JB . Factorial experiments. Am J Prev Med. 2014;47(4):498‐504. 25092122 10.1016/j.amepre.2014.06.021 PMC4171184

[65] Lin Y , Zhu M , Su Z . The pursuit of balance: an overview of covariate‐adaptive randomization techniques in clinical trials. Contemp Clin Trials. 2015;45(Pt A):21‐25. 10.1016/j.cct.2015.07.011 26244705

[66] Suresh K . An overview of randomization techniques: an unbiased assessment of outcome in clinical research. J Human Reproduct Sci. 2011;4(1):8‐11. 10.4103/0974-1208.82352 PMC3136079 21772732

[67] Vasilevsky NA , Brush MH , Paddock H , et al. On the reproducibility of science: Unique identification of research resources in the biomedical literature. PeerJ *,* 1, e148. 10.7717/peerj.148. eCollection 2013. PMID: 24032093 PMC3771067 24032093

[68] Bandrowski A , Brush M , Grethe JS , et al. The Resource Identification Initiative: a cultural shift in publishing. Brain Behav. 2016;6(1):e00417. 10.1002/brb3.417 PMC4834942 27110440

[69] Lazic SE . The problem of pseudoreplication in neuroscientific studies: is it affecting your analysis? BMC Neurosci. 2010;11(1):5. 10.1186/1471-2202-11-5 20074371 PMC2817684

[70] Strasak AM , Zaman Q , Marinell G , Pfeiffer KP , Ulmer H . The use of statistics in medical research: a comparison of the New England journal of medicine and nature medicine. Am Statistic. 2007;61(1):47‐55.

[71] Kimmelman J , Mogil JS , Dirnagl U . Distinguishing between exploratory and confirmatory preclinical research will improve translation. PLoS Biol. 2014;12(5):e1001863. 10.1371/journal.pbio.1001863 24844265 PMC4028181

[72] Nayak B , Hazra A . How to choose the right statistical test? Indian J Ophthalmol. 2011;59(2):85‐86. 21350275 10.4103/0301-4738.77005 PMC3116565

[73] Maxwell SE , Kelley K , Rausch JR . Sample size planning for statistical power and accuracy in parameter estimation. Annu Rev Psychol *,* 2008;59:537–563. 10.1146/annurev.psych.59.103006.093735, 1 17937603

[74] Button KS , Ioannidis JP , Mokrysz C , et al. Power failure: why small sample size undermines the reliability of neuroscience. Nat Rev Neurosci. 2013;14(5):365‐376. 10.1038/nrn3475 23571845

[75] Onwuegbuzie A , Leech N . Post hoc power: a concept whose time has come. Understanding Stat. 2004;3(4):201‐230.

[76] Goodman S , Berlin J . The use of predicted confidence intervals when planning experiments and the misuse of power when interpreting results. Ann Intern Med. 1994;121(3):200‐206. 8017747 10.7326/0003-4819-121-3-199408010-00008

[77] Levine M , Ensom MH . Post hoc power analysis: an idea whose time has passed? Pharmacotherapy. 2001;21(4):405‐409. 11310512 10.1592/phco.21.5.405.34503

[78] Wilkinson L , Inference TTF o S . Statistical methods in psychology journals: guidelines and explanations. Am Psychol. 1999;54(8):594‐604.

[79] Nuzzo R . Scientific method: statistical errors. Nature. 2014;506(7487):150‐152. 10.1038/506150a 24522584

[80] Lazic SE . Experimental design for laboratory biologists: Maximising information and improving reproducibility. Cambridge, UK: Cambridge University Press; 2016.

[81] Hoenig J , Heisey D . The abuse of power: the pervasive fallacy of power calculations for data analysis. Am Statistic. 2001;55(1):1‐6.

[82] Smith AH , Bates MN . Confidence limit analyses should replace power calculations in the interpretation of epidemiologic studies. Epidemiology. 1992;3(5):449‐452. 1391138 10.1097/00001648-199209000-00011

[83] Wilcox R . Introduction to robust estimation and hypothesis testing. 3 San Diego, CA: Academic Press; 2013.

[84] Frost J. 2015. Choosing between a nonparametric test and a parametric Test Retrieved from http://blog.minitab.com/blog/adventures‐in‐statistics‐2/choosing‐between‐a‐nonparametric‐test‐and‐a‐parametric‐test

[85] Rousselet GA , Foxe JJ , Bolam JP . A few simple steps to improve the description of group results in neuroscience. Eur J Neurosci. 2016;44(9):2647‐2651. 10.1111/ejn.13400 27628462

[86] Altman DG , Bland JM . Standard deviations and standard errors. BMJ. 2005;331(7521):903. 10.1136/bmj.331.7521.903 16223828 PMC1255808

[87] Nagele P . Misuse of standard error of the mean (SEM) when reporting variability of a sample. A critical evaluation of four anaesthesia journals. Br J Anaesth. 2003;90(4):514‐516. 12644429 10.1093/bja/aeg087

[88] Barde M , Barde P . What to use to express the variability of data: standard deviation or standard error of the mean? Perspect Clinic Res. 2012;3(3):113‐116. 10.4103/2229-3485.100662 PMC3487226 23125963

[89] Scargle J . Publication bias: the "file‐drawer" problem in scientific inference. J Scientif Explor. 2000;14(1):91‐106.

[90] Simmons JP , Nelson LD , Simonsohn U . False‐positive psychology: undisclosed flexibility in data collection and analysis allows presenting anything as significant. Psychol Sci. 2011;22(11):1359‐1366. 10.1177/0956797611417632 22006061

[91] Als‐Nielsen B , Chen W , Gluud C , Kjaergard LL . Association of funding and conclusions in randomized drug trials: Areflection of treatment effect or adverse events. JAMA. 2003;290(7):921‐928. 12928469 10.1001/jama.290.7.921

[92] Thompson D . Understanding financial conflicts of interest. N Engl J Med. 1993;329(8):573‐576. 8336759 10.1056/NEJM199308193290812

[93] Young S . Bias in the research literature and conflict of interest: an issue for publishers, editors, reviewers and authors, and it is not just about money. J Psychiatry Neurosci. 2009;34(6):412‐417. 19949717 PMC2783432

[94] Chambers C , Feredoes E , Muthukumaraswamy S , Etchells P . Instead of “playing the game” it is time to change the rules: registered reports at AIMS neuroscience and beyond. AIMS Neurosci. 2014;1(1):4‐17. 10.3934/Neuroscience2014.1.4

[95] Godlee F , Gale CR , Martyn CN . Effect on the quality of peer review of blinding reviewers and asking them to sign their reports: Arandomized controlled trial. JAMA. 1998;280(3):237‐240. 9676667 10.1001/jama.280.3.237

[96] Schroter S , Black N , Evans S , Carpenter J , Godlee F , Smith R . Effects of training on quality of peer review: randomised controlled trial. BMJ. 2004;328(7441):673. 10.1136/bmj.38023.700775.AE 14996698 PMC381220

[97] Smith R . Peer review: Aflawed process at the heart of science and journals. J R Soc Med. 2006;99(4):178‐182. 10.1258/jrsm.99.4.178 16574968 PMC1420798

[98] Tennant JP , Dugan JM , Graziotin D , et al. A multi‐disciplinary perspective on emergent and future innovations in peer review. F1000Res. 2017;6:1151. 10.12688/f1000research.12037.3 29188015 PMC5686505

